# Comparison of Functional Connectivity Estimated from Concatenated Task-State Data from Block-Design Paradigm with That of Continuous Task

**DOI:** 10.1155/2017/4198430

**Published:** 2017-01-16

**Authors:** Yang Zhu, Lin Cheng, Naying He, Yang Yang, Huawei Ling, Hasan Ayaz, Shanbao Tong, Junfeng Sun, Yi Fu

**Affiliations:** ^1^Department of Neurology, Shanghai Second People's Hospital, Shanghai 200011, China; ^2^School of Biomedical Engineering, Shanghai Jiao Tong University, Shanghai 200030, China; ^3^School of Biomedical Engineering, Science & Health Systems, Drexel University, Philadelphia, PA 19104, USA; ^4^Department of Radiology, Ruijin Hospital, School of Medicine, Shanghai Jiao Tong University, Shanghai 200025, China; ^5^Department of Neurology & Institute of Neurology, Ruijin Hospital, School of Medicine, Shanghai Jiao Tong University, Shanghai 200025, China

## Abstract

Functional connectivity (FC) analysis with data collected as continuous tasks and activation analysis using data from block-design paradigms are two main methods to investigate the task-induced brain activation. If the concatenated data of task blocks extracted from the block-design paradigm could provide equivalent FC information to that derived from continuous task data, it would shorten the data collection time and simplify experimental procedures, and the already collected data of block-design paradigms could be reanalyzed from the perspective of FC. Despite being used in many studies, such a hypothesis of equivalence has not yet been tested from multiple perspectives. In this study, we collected fMRI blood-oxygen-level-dependent signals from 24 healthy subjects during a continuous task session as well as in block-design task sessions. We compared concatenated task blocks and continuous task data in terms of region of interest- (ROI-) based FC, seed-based FC, and brain network topology during a short motor task. According to our results, the concatenated data was not significantly different from the continuous data in multiple aspects, indicating the potential of using concatenated data to estimate task-state FC in short motor tasks. However, even under appropriate experimental conditions, the interpretation of FC results based on concatenated data should be cautious and take the influence due to inherent information loss during concatenation into account.

## 1. Introduction

In the past few decades, fMRI has been widely used in various fields of brain science. The initial use of fMRI was to identify the brain regions related to specific mental behaviors by task-related activation analysis [1–3]. Furthermore, advances in fMRI have resulted in numerous studies focusing on temporal correlations between different brain regions and such correlations were interpreted as functional connectivity (FC) [4–8]. FC estimated from resting-state fMRI (i.e., no task engagement) is dominating this field [9–11]. Nevertheless, FC during task state is also a promising topic and has been used to investigate how task loads modulate functional brain organization [12–15]. For example, in working memory system, increasingly negative correlations emerged in the dorsal region of the posterior cingulate cortex during steady-state* N*-back task [16]. In motor system, the motor-related network was reported to be modulated during voluntary movements based on fMRI [12] and MEG [17]. In addition, the FC strength was related with the rate of finger tapping in key regions of the motor network [18]. In addition to working memory and motor systems, regions engaged in auditory [19] and visual [20, 21] functions also demonstrated modulated FC during corresponding tasks. These findings suggested that task-state FC is a promising method to investigate the task-induced modulations of functional brain networks and can be further applied to the research of neurological and psychological diseases.

Given the noisy and uncomfortable environment inside the fMRI scanner, it is not easy for the subjects, especially patients, to keep performing a task for several minutes without rest in order to acquire a continuous task suitable for traditional FC analysis. Thus, if it is possible to use block-design paradigms to collect the data and then estimate the task-state FC based on the concatenated task data from multiple task blocks, both activation analysis and FC analysis could be done based on the same block-design session, which would reduce the data collection time and simplify the procedures. In addition, more work regarding task-state FC could be done based on the existing large amount of block-design task datasets obtained in previous studies.

Studies with FC estimated from concatenated rest blocks of block-design fMRI experiment have been reported [22–24]. Salvador and colleagues reported that the concatenated rest blocks were able to yield similar correlation profiles to those of continuous rest data [25]. In addition, another study found similar correlation patterns between rest blocks of block-design data and continuous rest data for premotor cortex despite some differences [26]. In particular, Fair and colleagues found that the FC for the interleaved resting-state data taken from block-design task was both qualitatively and quantitatively similar to that of continuous rest data [22]. For task-state FC, it has been reported that the task-state FC and network were jointly shaped by the intrinsic network architectures represented by intrinsic resting-state FC and evoked network architectures represented by activation pattern [27–30], which makes task-state FC more complex than resting-state FC. Thus, there is no guarantee that data splicing, which did not induce significant differences between continuous resting-state FC and FC estimated from concatenated resting-state data, would not induce differences between task-state FC estimated from concatenated task blocks and continuous task data. To the best of our knowledge, the similarity of task-state FC between concatenated task blocks and continuous task data has not yet been examined, although studies have already used concatenated task blocks to estimate task-state FC and brain networks [31–35]. Furthermore, previous studies only examined the similarity of seed-based resting-state FC. Region of interest- (ROI-) based FC and brain network topology could provide more comprehensive information regarding the underlying brain activities during tasks. Therefore, in addition to seed-based FC, the similarities of ROI-based FC and brain network topology between concatenated task blocks and continuous task data are also worthy of investigation.

With the above considerations, blood-oxygen-level-dependent (BOLD) signals collected from 24 healthy subjects were used to make comparisons between concatenated task blocks and continuous task data from several perspectives, including ROI-based FC, seed-based FC, and brain network topology. In this study, the collected continuous task data was longer than the concatenated task blocks. Considering that data length was reported to be critical in FC estimation [36–39], we used a continuous task data segment with identical length to the concatenated task blocks in comparison to ensure that the results would not be affected by mismatched data length. Furthermore, as nonstationarity of FC across time has already been reported [37, 40, 41], comparisons were performed between concatenated task blocks and several continuous task data segments extracted by sliding window to test the robustness of comparisons regarding nonstationarity of FC. Our results are expected to provide suggestions regarding whether it would be appropriate to completely substitute continuous task data by concatenated task blocks.

## 2. Materials and Methods

### 2.1. Participants

Twenty-four healthy right-handed volunteers (male/female: 12/12; age: 53.8 ± 5.3 years) were enrolled in this study. All participants provided written informed consent and procedures were reviewed and approved by the Ethics Committee of Shanghai Second People's Hospital, Shanghai, China.

### 2.2. Experiment Design

The fMRI experiment for each subject consisted of two sessions. In the continuous task session, subjects were instructed to keep performing hand closing and opening (HCO) one time per second paced by the cues displayed on the screen with indicated hand for 4 minutes and 20 seconds. The second session was a block-design paradigm consisting of six rest blocks alternated with five HCO task blocks, preceded by 8 sec of preparing period. Each block lasted for 20 sec. At the rest block, subjects were instructed to remain motionless, relaxing, and awake; while in HCO task block, subjects were opening and closing the indicated hand one time per second paced by the cues. Fifteen subjects using their left hands in continuous task session and block-design session were regarded as left hand group (LHG). The other nine subjects who were using their right hands in continuous task session and block-design session were regarded as right hand group (RHG). Subjects were randomly instructed to use left/right hand. The whole experimental procedure was monitored by a physician to ensure that the subjects performed the task correctly.

### 2.3. Image Acquisition

All images were acquired by a 3.0 T Signa Excite Gemse MRI system (GE Healthcare, Milwaukee, WI, USA) at Ruijin Hospital. The head of the subject was snugly fixed by a foam pad to reduce head movements and motion-induced noise. 3D structural MRI was acquired from each subject using a T1-weighted MPRAGE sequence (TR = 5.6 ms; TE = 1.7 ms; flip angle = 12°; matrix size = 256 × 256; voxel size = 1 × 1 × 1 mm^3^), yielding 196 contiguous sagittal slices (1 mm thick) covering the whole brain. BOLD data were acquired with the same EPI sequence (TR = 2000 ms; TE = 30 ms; flip angle = 90°; matrix size = 64 × 64; voxel size = 3.75 × 3.75 × 4 mm^3^) for all subjects.

### 2.4. Data Preprocessing

For both continuous task session and block-design session, identical preprocessing procedures were performed using SPM8 (Wellcome Trust Centre for Neuroimaging, University College London, London, UK) and scripts from the DPARSFA toolbox [42]. The data of the first 8 sec (4 volumes) were abandoned to avoid the magnetization equilibrium effects and also allow the subjects to get ready for the experiments. The remaining fMRI data were spatially realigned to the mean image, slice-timing corrected using the middle slice as the reference frame, detrended, and high-pass filtered (>0.01 Hz). In addition, the fMRI data were coregistered with each subject's anatomical data. The anatomical images were then segmented [43]. Based on the deformation field obtained from segmentation step, the Automated Anatomical Labeling (AAL) atlas [44] with 116 ROIs and the motor-related key ROIs were warped to subject's native brain space and the representative BOLD time course for each ROI was estimated in the native brain space. After that, nuisance covariates including Friston 24 parameters of head motion [45], white matter, and cerebrospinal fluid signals were regressed out from the representative BOLD time courses. In addition, the autocorrelation was removed by a first-order autoregressive model using scripts from SPM8.

### 2.5. Extraction of Task Blocks Data

**Figure 1:**
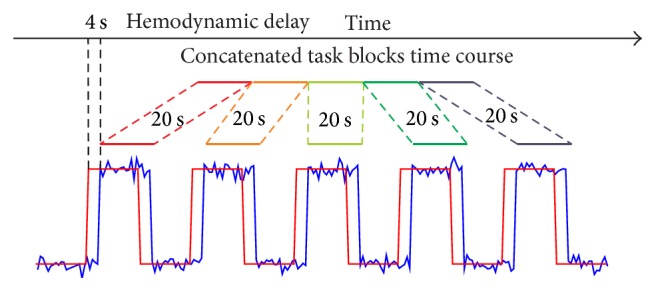
A schematic diagram for extracting task blocks from block-design session. A temporal shift of 2 volumes was adopted for the hemodynamic delay.

In order to estimate the FC based on concatenated task blocks, we extracted the task blocks from the block-design session. Due to neurovascular coupling, the hemodynamic response is delayed compared with neural activities. Thus, following previous study [22], each task block was shifted by 2 volumes; that is, two volumes immediately after the start of each task block were excluded and the starting two volumes of the following rest block were included. After that, the five task blocks were concatenated for further analysis (100 sec, 50 volumes; Figure 1). Note that the concatenated task blocks are referred to as concatenated data hereafter.

### 2.6. ROI-Based FC Analysis

In order to compare the ROI-based FC and the brain network topology based on continuous data with those estimated from concatenated data, ROI-based FC analysis was performed for both continuous data and concatenated data. In this study, the length of concatenated data was 100 sec (50 volumes), while the total length of continuous data collected was 250 sec. Considering that data length was reported to be critical in FC estimation and the comparison of functional connectivity should be performed under matching data lengths [36–39], we extracted continuous data segments of 100 sec for comparison so as to ensure that the comparison would not be affected by mismatched data length. In order to take the temporal nonstationarity of BOLD signal into account, four data segments of 100 sec were selected from the whole continuous data using sliding windows with 50% overlap and the four continuous data segments were denoted as continuous segments 1, 2, 3, and 4, respectively (i.e., continuous segment 1: 1–100 sec; continuous segment 2: 50–150 sec; continuous segment 3: 100–200 sec; continuous segment 4: 150–250 sec). For each subject, Pearson's correlation coefficients of all pairs between 116 ROI time courses were calculated and then were* r*-to-*z* transformed by Fisher's transformation, resulting in a 116 × 116 association matrix for each data segment, including concatenated data and the four continuous segments.

### 2.7. Topological Analysis of Brain Networks

In order to compare the brain network topology estimated from continuous segments and concatenated data, the association matrices were converted into adjacency matrices by eliminating the entries, which were smaller than the preset thresholds. The thresholds were selected according to the predefined sparsity, which was defined as the ratio of the existing edge number over the maximum possible number of edges in a network. In order to eliminate the effects of the selection of sparsity, adjacency matrices with sparsity ranging from 0.1 to 0.5 with a sparsity increment of 0.02 for each association matrix were examined. The selection of sparsity range was based on two considerations, which were the following: (1) the average degree of the functional brain networks should not be smaller than 2 × ln⁡(*N*) to apply graph theory [46]; (2) in order to reduce the false discovery rate, the sparsity should be as small as possible [24]. Based on the adjacency matrices, clustering coefficient (CC) and characteristic path length (Lambda), which were widely used to evaluate the functional segregation and integration of brain networks, were calculated for both concatenated data and continuous segments [47, 48]. In order to account for the variability across different scanning sessions and subjects, the CC and Lambda were normalized by comparing the values of parameters with the corresponding average values across 50 random graphs with preserved degree distribution [49].

### 2.8. Seed-Based FC Analysis

In addition to the ROI-based FC and brain network topology, the continuous segments and concatenated data were also compared from the perspective of seed-based FC. Since subjects were performing HCO during continuous task session and task blocks, seed ROIs were defined as spheres of radius of 20 mm centered on coordinates *x* = ±38, *y* = −26, and *z* = 56, representing contralateral hand part of primary motor area (M1) for LHG and RHG, respectively [50, 51]. After that, a voxel-wise FC map (covering all voxels of brain) was created for the corresponding seed ROI by calculating Pearson's correlation coefficient (*r*-to-*z* Fisher's transformation applied) between the seed time course and the time course of each voxel for both concatenated data and continuous segments of each subject.

### 2.9. Comparison between Continuous Data and Concatenated Data

To examine whether the concatenated data was able to provide similar information as the continuous data, comparisons were performed between each continuous segment and concatenated data from perspectives of ROI-based FC, the brain network topology, and the seed-based FC map. For ROI-based FC, three measurements were estimated for comparison. The first one was the number of significantly shifted ROI-based FCs obtained by paired *t*-test between continuous segment and concatenated data. The second one was the similarity (measured through Pearson's correlation) of association matrices between continuous segment and concatenated data. The last one was the overlap ratios of adjacency matrices under ranging sparsity between continuous segment and concatenated data. The overlap ratio was defined as the ratio of the number of overlapped FCs between two data segments over the total number of FCs under specific sparsity. With respect to brain network topology, comparison was performed by paired *t*-test between continuous segment and concatenated data for normalized CC and Lambda under ranging sparsity. For seed-based FC, paired *t*-test was performed to identify voxels with significantly changed FC to the corresponding seed. In addition, the similarity of seed-based FC maps between continuous segment and concatenated data was estimated through Pearson's correlation for each subject.

In order to examine whether the similarity between concatenated data and continuous data was high enough, a reference of similarity is needed. If the similarity between the concatenated data and continuous segment is at the same level of the reference of similarity, it could be concluded that the continuous data can be substituted by concatenated data in FC analysis. The optimal reference of similarity would be the similarity of continuous segment with another data segment selected from the same session, as they are assumed to be statistically interchangeable in fMRI studies. Thus, a 100 sec-long reference continuous segment was extracted from the continuous task session to calculate the reference of similarity. Since four continuous segments were used in comparison, four reference continuous segments were extracted correspondingly. There were two criteria for the selection of reference continuous segments, which were the following: (1) there should be no overlap between the continuous segment and corresponding reference continuous segment; (2) the reference continuous segment should be temporally farthest away from continuous segment. Thus, continuous segment 4 was regarded as the reference continuous segment for both continuous segments 1 and 2, while continuous segment 1 was regarded as the reference continuous segment for both continuous segments 3 and 4. The similarity between each pair of reference continuous segment and continuous segment was calculated in terms of association matrices, overlap ratios of adjacency matrices, and seed-based FC maps and further compared with the similarities between corresponding continuous segment and concatenated data.

In addition, estimation of FC is sensitive to data length as the variance increases when data length shrinks [36–39]. Therefore, we also performed comparisons between reference pair and comparison pair with data segments of 80 sec (40 volumes) and 60 sec (30 volumes), respectively, for ROI-based FC and seed-based FC to see whether the data length would affect our findings. For data segments of 80 sec, five continuous segments and five reference continuous segments were extracted from continuous task data, respectively. With respect to data segments of 60 sec, seven continuous segments and seven reference continuous segments were extracted, respectively. The reference continuous segments were selected according to the two criteria used above.

## 3. Results

### 3.1. ROI-Based FC

**Figure 2:**
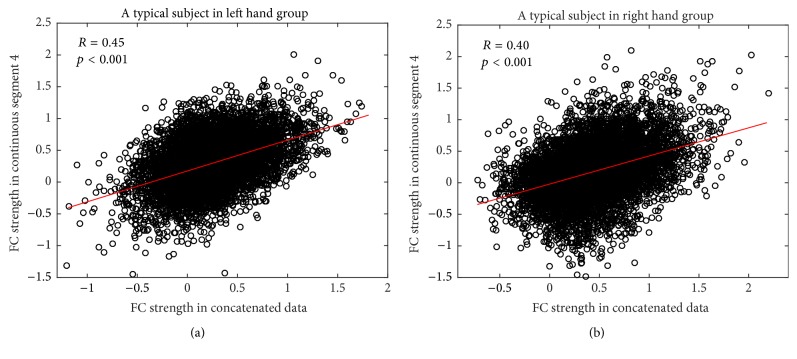
Scatter plots of FC strength for continuous segment 4 and concatenated data for a typical subject in LHG (a) and a typical subject in RHG (b). There are a total of 6670 FCs between 116 nodes (i.e., ROIs), and each circle represents one FC.

**Figure 3:**
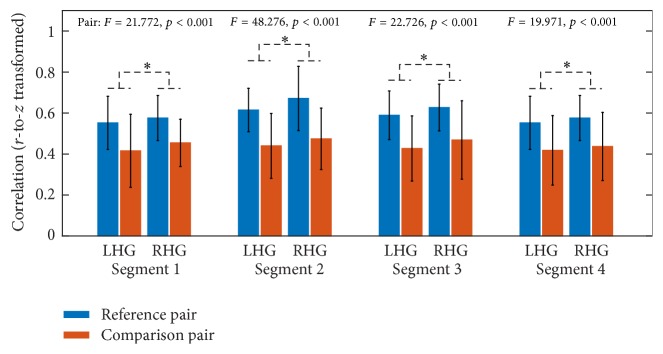
The similarities of association matrices (*r*-to-*z* transformed) in comparison pair as well as those in reference pair in LHG and RHG for four continuous segments. Error bars denote the standard deviation across subjects.

With respect to ROI-based FC, paired *t*-tests were performed to identify significantly changed FC between continuous segments and concatenated data. With respect to all four continuous segments, the comparison between continuous segment and concatenated data resulted in no significantly changed FC for both LHG and RHG after multiple comparison correction by FDR. In addition, the similarity of association matrices between continuous segment and concatenated data was estimated for each subject. For all subjects in LHG and RHG, the association matrices of all four continuous segments were significantly correlated with those of concatenated data (*p* < 0.001). Hereafter, the illustrations of results were mainly based on continuous segment 4.  Figure 2 illustrates the results of correlation analysis based on continuous segment 4 for a typical subject in LHG (Figure 2(a)) and a typical subject in RHG (Figure 2(b)). However, by comparing the similarity of comparison pair (i.e., continuous segment and concatenated data) with that of reference pair (i.e., continuous segment and reference continuous segment), mixed ANOVA with one within-subject factor PAIR (comparison pair versus reference pair) and one between-subject factor GROUP (LHG versus RHG) suggested that the similarity of reference pair was significantly greater than that of comparison pair for all four continuous segments in both groups (Figure 3).

**Figure 4:**
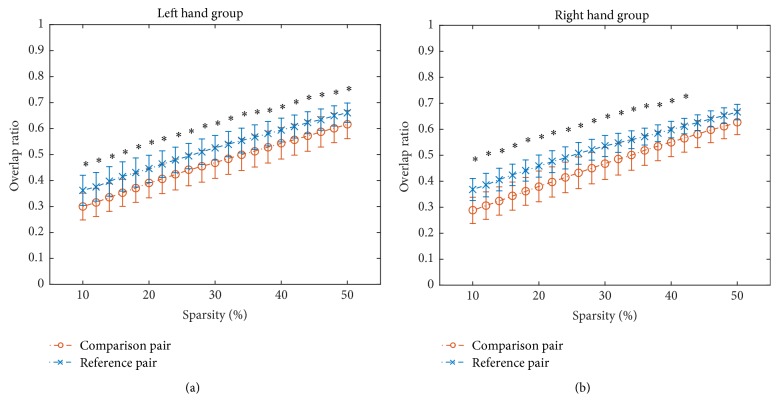
The overlap ratios of adjacency matrices for comparison pair as well as those for reference pair under sparsity from 0.1 to 0.5 with an increment of 0.02 in LHG (a) and RHG (b) for continuous segment 4. Error bars denote the standard deviation across subjects. Asterisk (*∗*) denotes that the overlap ratio for reference pair is significantly greater (FDR corrected, *p* < 0.05) than that for comparison pair under the sparsity.

With respect to the overlap ratios of adjacency matrices, the overlap ratios in reference pair were significantly greater (FDR corrected, *p* < 0.05) than those in comparison pair under all ranging sparsities in LHG (Figure 4(a)) and most ranging sparsities in RHG (Figure 4(b)).  Figure 4 illustrated the results of continuous segment 4 and the results of other three continuous segments were similar.

### 3.2. Brain Network Topology

**Figure 5:**
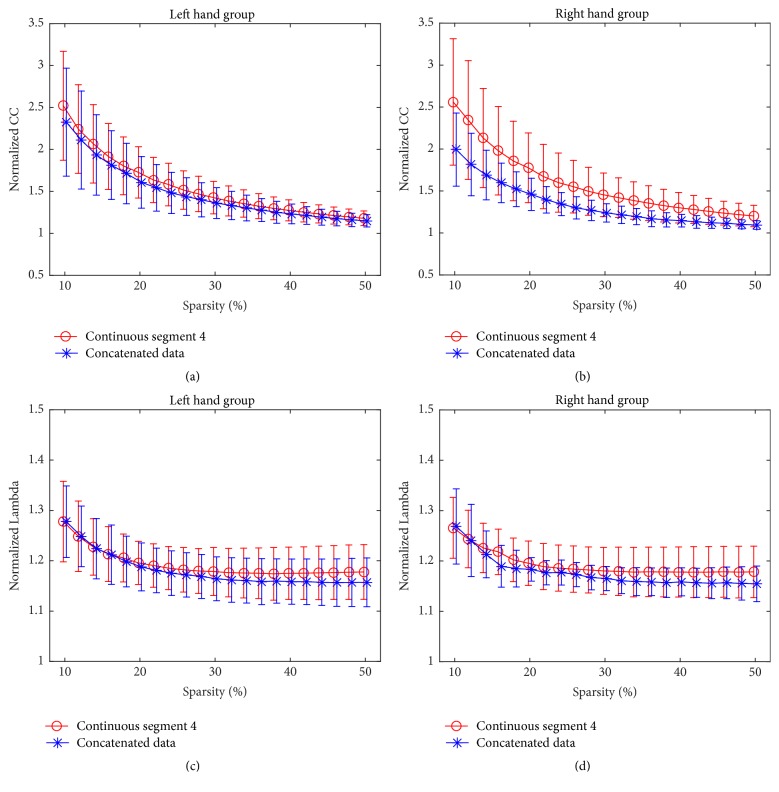
The normalized CC and Lambda of continuous segment 4 and concatenated data in LHG ((a) and (c)) and RHG ((b) and (d)), respectively. Error bars denote the standard deviation across subjects.

For brain network topology, normalized CC and Lambda of brain networks were compared between continuous segments and concatenated data in both groups, respectively. No significant difference was found for both measures at sparsity ranging from 0.1 to 0.5 for continuous segment 4 (Figure 5), and the results of other three continuous segments were similar, which suggested that there was no substantial difference of network topology between continuous data and concatenated data.

### 3.3. Seed-Based FC Map

**Figure 6:**
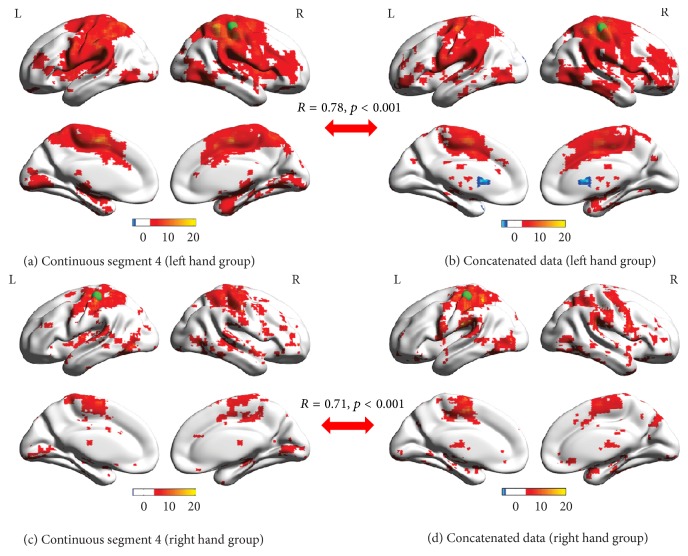
Group-level seed-based FC maps of continuous segment 4 ((a) and (c)) and concatenated data ((b) and (d)) in LHG ((a) and (b)) and RHG ((c) and (d)). The index of color bar indicates* t* value of *t*-test. Voxels with hot color have positive FC with corresponding seed region (*x* = ±38, *y* = −26, and *z* = 56), indicated by a green sphere, with cool color for negative FC. *R* values and *p* values ((a) versus (b) and (c) versus (d)) indicate that the similarities between the seed-based FC maps of continuous segment 4 and concatenated data are significant.

For both LHG and RHG, one group-level seed-based FC map was obtained by one-sample *t*-test (FDR corrected, *p* < 0.05) for continuous segments (Figures 6(a) and 6(c) illustrated the FC map of continuous segment 4) and concatenated data (Figures 6(b) and 6(d)). In addition, paired *t*-tests were used to identify the differences of seed-based FC maps between continuous segments and concatenated data and no voxel with significantly changed FC with seed ROI was identified after multiple comparison correction (FDR corrected, *p* < 0.05) in any of the four continuous segments. In addition, the similarity of group-level seed-based FC maps between continuous segment 4 and concatenated data as calculated by Pearson's correlation was significant for both LHG (*R* = 0.78; *p* < 0.001) and RHG (*R* = 0.71; *p* < 0.001).

**Figure 7:**
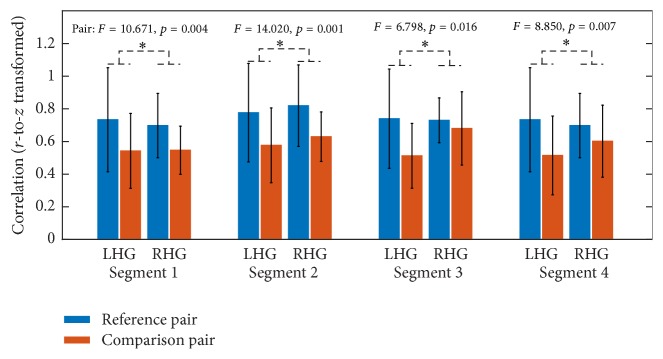
The similarities of seed-based FC maps (*r*-to-*z* transformed) in comparison pair as well as those in reference pair in LHG and RHG for four continuous segments. Error bars denote standard deviation across subjects.

In addition to the similarity between group-level seed-based FC maps, the similarities of seed-based FC maps in comparison pair were compared with similarities of the reference pair for four continuous segments. For all four continuous segments, mixed ANOVA suggested that the similarities in reference pair were significantly greater than those in comparison pair in both groups (Figure 7).

### 3.4. Comparisons under Different Data Segment Length

Mixed ANOVA was also used to examine the main effects of within-subject factor PAIR (comparison pair versus reference pair) for data segments of 60 sec and 80 sec, respectively. These results, as well as the case of 100 sec, are listed in Table 1. For ROI-based FC, just like the results based on data length of 100 sec, the similarity in comparison pair was significantly smaller than reference pair for most continuous segments in both groups under data segment lengths of 60 sec and 80 sec, respectively (Table 1). However, for seed-based FC, no significant differences between comparison pair and reference pair were identified for most continuous segments for these data lengths (Table 1).

## 4. Discussion

In this study, we compared the concatenated data with continuous segments from the perspectives of ROI-based FC, seed-based FC, and brain network topology to investigate whether it would be appropriate to substitute continuous task-state data by concatenated task-state data when performing FC analysis.

With respect to the ROI-based FC, no significantly changed FC was found between continuous segments and concatenated data for both groups. In addition, there were no differences between seed-based FC maps of continuous segments and concatenated data. When considering the brain network topology, the normalized CC and Lambda of concatenated data were not significantly different from those of continuous segments for both groups (Figure 5). These results suggested that the concatenated data was not significantly different from the continuous segments in terms of ROI-based FC, seed-based FC, and brain network topology, indicating the feasibility of using concatenated data to estimate FC and brain network statistics, which is in line with studies simulating continuous resting-state data by concatenated resting-state blocks [22, 23]. Previous studies reported that the intrinsic network architectures as represented by intrinsic brain activities and evoked network architectures represented by activation pattern jointly shaped the task-state FC and network organization [27–30]. Therefore, the lack of significant differences between continuous segments and concatenated data suggested that the splicing might not dramatically affect the expressions of intrinsic network architecture as well as evoked network architectures, and the information lost during splicing would not result in significant distortions in terms of ROI-based FC, seed-based FC, and brain network topology.

However, a lack of significant differences between continuous segments and concatenated data does not necessarily suggest that continuous data can be completely substituted with concatenated data in performance of FC analysis without taking the influences of concatenation into account. According to the correlation analysis between continuous data and concatenated data, though the correlation is significant (Figure 2), we cannot draw conclusions from it alone as it may only indicate that overall similarities of network architectures and the significant correlations observed might instead be due to a large number of data points. In order to further test whether the continuous data can be substituted by concatenated data, we extracted another reference continuous segment from the same session under the assumption that two data segments from one session are statistically interchangeable. Our results demonstrated that the similarities in comparison pair were significantly smaller than those in reference pair in terms of association matrices (Figure 3), overlap ratios of adjacency matrices (Figure 4), and seed-based FC maps (Figure 7). So, even though the information lost during splicing did not dramatically affect expressions of intrinsic and evoked network architectures, its influences on expressions of network architectures still existed. Three arguments may account for the difference between comparison pair and reference pair. (1) The influences might be due to the formation of the concatenated data. It has been reported that the task-state network and FC were jointly shaped by the intrinsic network architectures represented by intrinsic brain activities and evoked network architectures represented by activation pattern [27–30]. The extracted task blocks used in this study were only 20 sec long, which inherently ignores the information in low frequency range, where intrinsic brain activities were prominent [4, 9]. Thus, the information loss of intrinsic brain activities due to splicing would likely affect the expression of intrinsic network architectures and further resulted in the reduced similarity in comparison pair than in reference pair. (2) In addition, since the reference pair was extracted from the same session, while the comparison pair was from two separate sessions, intersession differences might also contribute to the reduced similarity of comparison pair [52, 53]. (3) Another cause of reduced similarity of comparison pair may be distinct conditions of subjects between continuous task and block-design task. The continuous task was relatively long, which might cause tiredness and inattention of subjects, while the block-design with shorter task blocks and rest blocks would allow subjects to recover and keep attention.

Numerous studies have reported the nonstationarity of FC and network architectures, which are subject to change over time [37, 40, 41]. Our study shows that data segments of reference pair had differences in terms of association matrices, overlap ratios of adjacency matrices, and seed-based FC maps, which are consistent with the findings of above studies. Taking the nonstationarity of FC into account, we also performed comparisons based on the four continuous segments, which were generated by sliding window with 50% overlap. For all the continuous segments, the results were similar, suggesting that our findings were robust to the nonstationarity of FC during continuous data. With respect to data segment length, the inconsistent results of seed-based FC under data segment length of 60 sec and 80 sec compared with those under data segment length of 100 sec might be due to the increased variance introduced by shortened data lengths. Note that the seed-based FC was obtained by voxel-wise estimation, which is more susceptible to this increased variance, while estimation of ROI-based FC is based on ROI-representative BOLD time series obtained by averaging the voxel BOLD time series within the ROI, which reduces the noise of the representative BOLD time series and therefore offsets the increasing of variance. Therefore, the results of ROI-based FC under data length of 60 sec and 80 sec were consistent with those under data length of 100 sec, while the results of seed-based FC were not.

In addition to concatenation, previous studies also extracted resting-state activities from block-design task data by adopting the general linear model (GLM) to regress out the effect of task and estimate the resting-state FC using the residuals. However, the GLM method was not observed to be as effective as the concatenation method according to the results of Fair et al. [22]. The authors attributed this difference to the nonlinearity of task effects on the intrinsic brain activities and thus the GLM was not able to effectively remove the task effects [20, 54]. For the application of GLM method on task-state FC estimation based on block-design task data, another shortcoming was that task-state FC and network were reported to be jointly shaped by the intrinsic network architectures represented by intrinsic resting-state FC and evoked network architectures represented by activation pattern [27–30]. Thus, using GLM to extract only the evoked brain activities from block-design task data to estimate task-state FC would ignore the intrinsic network architectures, resulting in significant differences from the task-state FC based on continuous task data. Therefore, it was reported that the concatenation was a superior method to estimate task-state FC based on block-design task data.

There were several limitations of this study. First, the time length of block-design session may not have been sufficient, though previous studies suggested that 100 sec was long enough to estimate reliable FC [37, 39]. Longer block-design session would result in longer concatenated data, which would enable a more comprehensive comparison of FC. Second, our study mainly focused only on the feasibility of concatenation in a motor task, which limited the direct interpretation of our results to motor function. More studies with tasks involving function of other cognitive domains should be done to verify whether a similar procedure can be validated for use in other tasks.

## 5. Conclusions

In this study, concatenated data were compared with continuous data in perspectives of ROI-based FC, seed-based FC, and brain network topology during a short motor task. According to our results, the concatenated data were not significantly different from the continuous data in multiple respects, indicating the potential of using concatenated data to estimate FC and brain networks for short motor tasks. However, since the similarities in comparison pair were significantly smaller than those in reference pair in terms of association matrices, overlap ratios of adjacency matrices, and seed-based FC maps, the interpretation of FC results based on concatenated data should be done cautiously and taking the influences caused by concatenation and the task type employed into account.

## Figures and Tables

**Table 1:** Main effect of PAIR (comparison pair versus reference pair) of mixed ANOVA used in similarities comparison of ROI-based FC and seed-based FC.

Data length	Segment number	ROI-based FC		Seed-based FC	
		*F* value	*p* value	*F* value	*p* value
50 TRs (100 s)	1	21.772	<0.001^*∗∗∗*^	10.671	0.004^*∗∗*^
	2	48.276	<0.001^*∗∗∗*^	14.020	0.001^*∗∗*^
	3	22.726	<0.001^*∗∗∗*^	6.798	0.016^*∗*^
	4	19.971	<0.001^*∗∗∗*^	8.850	0.007^*∗∗*^

40 TRs (80 s)	1	7.620	0.011^*∗*^	4.690	0.041^*∗*^
	2	16.659	<0.001^*∗∗∗*^	4.225	0.052
	3	17.730	<0.001^*∗∗∗*^	3.696	0.068
	4	10.880	0.003^*∗∗*^	2.520	0.127
	5	15.341	0.001^*∗∗*^	4.372	0.048^*∗*^

30 TRs (60 s)	1	1.768	0.197	5.783	0.025^*∗*^
	2	3.445	0.077	2.427	0.134
	3	9.656	0.005^*∗∗*^	1.834	0.189
	4	12.651	0.002^*∗∗*^	2.226	0.150
	5	5.248	0.032^*∗*^	3.443	0.077
	6	5.299	0.031^*∗*^	1.736	0.201
	7	9.734	0.005^*∗∗*^	2.004	0.171

^*∗∗∗*^ *p* < 0.001, ^*∗∗*^*p* < 0.01, and ^*∗*^*p* < 0.05.
